# High prevalence of group B streptococcus ST17 hypervirulent clone among non-pregnant patients from a Hungarian venereology clinic

**DOI:** 10.1186/s12879-019-4626-7

**Published:** 2019-11-28

**Authors:** Szilvia Kardos, Adrienn Tóthpál, Krisztina Laub, Katalin Kristóf, Eszter Ostorházi, Ferenc Rozgonyi, Orsolya Dobay

**Affiliations:** 1 Institute of Medical Microbiology, Semmelweis University, Nagyvárad tér 4, Budapest, H-1089 Hungary; 2 Institute of Laboratory Medicine, Semmelweis University, Budapest, Hungary; 3 Department of Dermatology, Dermatooncology and Venerology, Semmelweis University, Budapest, Hungary

**Keywords:** GBS, Serotypes, Surface proteins, Hypervirulent ST-17 clone, Macrolide resistance

## Abstract

**Background:**

Although *Streptococcus agalactiae* is the leading causative agent of neonatal sepsis and meningitis, recently it is increasingly isolated from non-pregnant adults. The relation between its presence in the genitourinary tract and manifested clinical symptoms of STD patients remains an open question. In this study, a complex epidemiological investigation of GBS isolates from a venerology clinic was performed.

**Methods:**

Ninety-six GBS isolates were serotyped and their genetic relatedness determined by PFGE. MLST was also performed for a subset of 20 isolates. The antibiotic susceptibility was tested with agar dilution. Surface proteins and the ST-17 hypervirulent clone was detected by PCR.

**Results:**

The serotype prevalence was the following: V (29.2%), III (27.1%), Ia (22.9%), IV (10.4%), II (5.2%) and Ib (4.2%). A strong association was demonstrated between surface protein genes and serotypes. All isolates were fully susceptible to penicillin, but erythromycin and clindamycin resistance was high (41.7 and 35.4%, respectively), and 8 phenotypically macrolide sensitive isolates carried the *ermB* gene.

21.9% of all strains belonged to the hypervirulent ST17 clone, most being of serotype III and all were *rib* +. We found a few serotype IV isolates belonging to several STs and one serotype V/ST110 strain, containing a 44-bp deletion in the *atr* allele.

**Conclusions:**

The presence of silent *ermB* genes is of worry, as their expression upon macrolide exposure could lead to unforeseen therapeutic failure, while clindamycin is used for intrapartum antibiotic prophylaxis, in case of penicillin allergy. The other alarming result is the high prevalence of ST17 among these strains from STD patients, who could be sources of further infections.

This is the first report from Hungary providing both serotyping and genotyping data of GBS isolates. These results could be helpful for vaccine production as the major vaccine candidates are capsular antigens or surface proteins.

## Background

*Streptococcus agalactiae* (or group B streptococcus, GBS) was for long thought to be an animal pathogen especially responsible for mastitis of cows, however, since the 1970’s it has been known as the leading pathogen of neonatal sepsis and meningitis [1, 2]. Still, nowadays, it ranks as the number one infectious cause of neonatal mortality in developed countries [3]. This bacterium colonizes the genital and gastrointestinal tract of healthy females in varying percentages. Colonization becomes more frequent in late adolescence and it can reach 10–40% among pregnant women [4]. The presence of this bacterium in the genital flora during pregnancy is the predominant risk factor for the development of invasive neonatal disease [5].

Moreover, increasing importance of GBS in infections of pregnant women or non-pregnant adults, especially the elderly or those with underlying diseases, is reported worldwide [3, 6, 7]. The incidence of GBS infections increases with age, most infections seen in the >65 years age category [6, 8]. These include skin infections, pneumonia, meningitis, endocarditis. The latter diseases are rarer, but are associated with high mortality: case fatality is considerably higher in adults than in neonates [7, 9, 10].

There are contradictory opinions in the literature whether the presence of GBS in the vaginal flora can be responsible for clinical symptoms. For example, Honig et al. failed to detect any correlation between vaginal colonization (even heavy colonization) and vaginal symptoms in a Rotterdam STD clinic [11]. On the other hand, others found that all examined GBS positive women had vaginal soreness and/or discharge and suggested that streptococci mostly play a secondary role and colonize an already damaged genital epithelium [12]. A very recent Hungarian study examined 100 removed intrauterine devices (57% of the patients showing some type of clinical symptom) and identified the presence of *S. agalactiae* in 14 cases [13].

Some authors suggest that GBS can be transmitted sexually as higher colonization rates were observed among STD patients, even from the male urethra [14], or among sexually active young people in a dormitory compared to the sexually inexperienced participants [15]. But again, in the above-mentioned study [11], no correlation was found between GBS colonisation and history of gonorrhea, chlamydia, genital herpes or genital warts. Similarly, Brazilian authors concluded that sexual contact did not seem to be the principal way of transmitting GBS among HIV positive women [16].

The most important virulence factor of *S. agalactiae* is its polysaccharide capsule, which is helpful for the bacterium in evading host defence mechanisms [7, 17]. To date, ten serotypes are distinguished based on the capsule (Ia, Ib, II, III, IV, V, VI, VII, VIII and IX). Surface proteins also play an important role in the pathogenesis of GBS [18]. The best-characterised antigens belong to the alpha-like protein family and are called Alpha-C protein, Epsilon (Alp1), Alp2, Alp3, Alp4, and Rib [19, 20]. They are encoded by mosaic genes and contain large internal tandem repeats, therefore exhibit size variations [21]. Usually every GBS isolate possesses one Alp-like gene, even if it is not expressed on the surface [20, 22]. These surface proteins show strong association with serotypes [20–24].

The majority of LOD (late onset disease) and a substantial proportion of EOD (early onset disease), with a high mortality rate in neonates have been associated with serotype III world-wide [7, 17, 25]. Different genotyping methods have revealed only a few genetic lineages among serotype III isolates [26–28]. Within the clonal complexes identified by MLST (multi-locus sequence typing), particularly one, ST-17 was shown to be associated with invasive neonatal infections, especially meningitis, therefore was called the “hypervirulent clone”.

Several further surface-exposed virulence factors have been identified among *S. agalactiae* isolates, which contribute to the enhanced adhesive properties of this bacterium. A highly prevalent cell-wall anchored protein, Gbs2018 was characterised first by Lamy et al. in 2006 [28], which has two major variants. Gbs2018C (later called as HvgA, hypervirulent GBS adhesion) was shown to be strictly specific for ST-17 [28, 29]. This variant can be responsible for the stronger intestinal colonization of the ST-17 isolates, as well as for crossing the intestinal and blood-brain barriers, leading to meningitis in increased rates [29]. The other variant, Gbs2018A (also called BibA, GBS immunogenic bacterial adhesin) was shown to be widely distributed among GBS isolates, representing several different serotypes [28, 30] and it also provides better survival in human blood and enhanced adherence to epithelial cells.

As it was underlined above, the pathogenic role of GBS colonization in STD patients remains an open question. The aim of the present study was to examine *S. agalactiae* isolates from non-pregnant adults, mostly patients of an STD ambulance. The complex epidemiological survey included serotyping, detection of surface proteins, PCR detection of the ST-17 hypervirulent clone, PFGE and MLST of representative isolates and antibiotic susceptibility testing.

## Methods

### Bacterial isolates

Ninety-six randomly selected GBS strains, deriving from the Department of Dermatology, Dermatooncology and Venerology, Semmelweis University, Budapest, over a 1-year period (October 2010 – September 2011), were involved in the study. During the study period, a total of 10,519 specimens arrived at the laboratory. Out of these, 296 were *S. agalactiae*, which equals to a 2.8% prevalence. Among the urogenital specimens (*n* = 4327), 259 were positive for *S. agalactiae* (6.0%), while among the dermatology specimens (*n* = 6192), only 37 were positive for *S. agalactiae* (0.6%).The vast majority of the examined isolates (*n* = 76) derived from the STD ambulance (which examines patients from the whole country), 14 from the general ambulance, six from the psoriasis ambulance. Most specimens were either vaginal (*n* = 38), urethral (*n* = 30), or from skin (*n* = 13); the others included glans (*n* = 8), urine (*n* = 4), and one each from nose, anus and ejaculate. The etiological role of GBS in the skin infections is more likely, however, the pathogenic role of the bacterium in STD patients remains unclear, especially if other microbes were simultaneously detected. The age of patients ranged between 15 and 77 years, with a mean age of 42.4 years. The genders were nearly equalised: 50 females and 46 males.

The following bacteria were also isolated from the patients: Ureaplasma spp.: *n* = 29, *Chlamydia trachomatis* D-K serotypes: *n* = 12, *Mycoplasma hominis*: *n* = 5, Ureaplasma / Mycoplasma: *n* = 1. There was no ongoing *Treponema pallidum* infection, but eight patients had syphilis treatment in the anamnesis. Every patient was negative for HIV, HBV, and HCV; HSV1–2 was not tested. In the urogenital specimens, the following Candida species were identified: *C. albicans* (*n* = 11), *C. krusei* (*n* = 3), *C. glabrata* (*n* = 3), *C. tropicalis* (*n* = 1). No yeasts were found in the dermatological samples. Trichomonas was present in three cases.

Identification of *S. agalactiae* was primarily done with routine laboratory tests (β-haemolysis, catalase negativity), followed by the Lancefield grouping with the Pastorex STREP agglutination Kit (BioRad, California, US). The identity of the isolates was confirmed in every case by PCR detection of the species-specific *dltR* gene, as described by Lamy et al. [28].

### Antibiotic susceptibility testing

Susceptibility to six antibiotics (penicillin, erythromycin, clindamycin, levofloxacin, moxifloxacin, and tetracycline) was done by agar dilution method, using an A400 multipoint inoculator (AQS Manufacturing Ltd., Southwater, UK), according to the EUCAST guidelines [31]. As controls, *Streptococcus agalactiae* ATCC 80200 and *S. pneumoniae* ATCC 49619 were used. The macrolide resistance genes *ermB*, *ermTR*, *mef,* and *linB* were detected by PCR [32–34]. The distinction between *mefA* and *mefE* was carried out by *BamHI* digestion, which generates two fragments in *mefA*, but none in *mefE*, as described before for *S. pneumoniae* [35].

Inducible clindamycin resistance was tested with the double-disc method, using 15 μg erythromycin and 2 μg clindamycin discs, as described by EUCAST [31], for those isolates which were resistant to erythromycin but had low clindamycin MIC.

### Serotyping

To distinguish first serotypes I, II and III, the Pastorex Strepto BI, BII, BIII latex agglutination test (Bio-Rad) was used. Subsequently, the serotype of every isolate was determined by multiplex PCRs for serotypes Ia-IV and V-VIII [36] and a separate PCR was used for serotype IX [37].

### Detection of surface proteins

The genes of Alpha-C, Alp2/3, Alp4, Rib and Epsilon proteins were detected by multiplex PCR, using a universal forward primer and a type-specific reverse primer for each protein, as described by Creti et al. [20].

### PCR detection of the cell-wall anchored protein variants Gbs2018A (BibA) and Gbs2018C (HvgA)

The strict specificity of the *hvgA* gene to the hypervirulent ST-17 MLST clone enables the rapid and cheap PCR detection of this clone instead of performing the real MLST [28].

A 345 bp fragment was amplified with the primers described by Tazi et al. [29]. The other variant of the same protein, Gbs2018A (*bibA*) was also detected by PCR [30].

### DNA isolation for the PCR reactions

In all cases, DNA was prepared by the ZR Fungal/Bacterial DNA MiniPrep (Zymo Research Corp., Irvine, CA, US). Briefly, approximately 100 mg bacterial cells (equivalent to 1–2 plateful ON cultures) were resuspended in 200 μl saline buffer and transferred to a specific Lysis Tube, containing beads. After 750 μl Lysis solution was added, the suspension was rigorously vortexed for 5 min on full speed, in order to disrupt the bacterial cell wall by bead beating. The mixture was then centrifuged at 10,000 g and the supernatant additionally filtered to remove cell debris. The filtrate – containing the DNA – was then purified by the usual column-binding method, also provided in the kit.

### Pulsed-field gel electrophoresis (PFGE)

To determine the genetic relatedness of the isolates, pulsed-field gel electrophoresis (PFGE) was used, as described by Benson et al. [38]. The complete bacterial genome was embedded in agarose plugs and lysed in several steps (with lysozyme, mutanolysin and proteinase K) to purify DNA. For the digestion, *Sma* I restriction enzyme was used (3 h, 25 °C). The digested samples were run in a 1% agarose gel along with N0340S Lambda Ladder PFG Marker (New England Biolabs, Hitchin, Hertfordshire, UK), in a Bio-Rad CHEF-DR® II PFGE machine, for 21 h at 14 °C, with the following pulse times: block 1, 5 s/15 s for 10 h, and block 2, 15 s/60 s for 11 h. After the gel image was captured, dendrograms were created by the Fingerprinting II software (Bio-Rad, Marnes-la-Coquette, France) and the PFGE patterns were analysed according to the criteria of van Belkum et al. [39].

### Multi-locus sequence typing (MLST)

Multi-locus sequence typing (MLST) was performed on 20 representative isolates, based on the PFGE patterns and the serotype - surface protein combinations. Well-defined sections of seven housekeeping genes (*adhP*, *pheS*, *atr*, *glnA*, *sdhA*, *glcK*, *tkt*) were amplified by PCR, using the primers provided by the MLST website [40]. The products were purified by the QIAquick PCR purification kit (QIAGEN, Hilden, Germany) and sent for sequencing to BIOMI Ltd., Gödöllő, Hungary. The allele sequences were compared to the MLST database and the sequence types were identified.

For statistical analysis, Fisher’s exact test or Chi-square test was applied.

## Results

### Antibiotic susceptibility

All isolates were fully susceptible to penicillin, and only three isolates were resistant to levofloxacin and moxifloxacin (Table 1). On the other hand, 82.3% of them were resistant to tetracycline, and the macrolide and lincosamide resistance was also high (41.7% to erythromycin and 35.4% to clindamycin). Among the 40 erythromycin-resistant strains, 27 possessed the *ermB* gene alone, two had *ermTR* alone, four had *ermB* and *ermTR* together, one carried *ermB* + *linB* together, and finally six had *mef* genes (five *mefE* and one *mefA*). Among the six isolates showing resistance to erythromycin, but sensitivity to clindamycin, the D-test was positive in four cases (meaning inducible MLS_B_ resistance), out of which two isolates expressed high-level (128 and 256 mg/L) and two isolates only low-level (16 and 32 mg/L) erythromycin resistance. These latter two isolates were *mef*-negative but possessed the *ermTR* gene. The association between M phenotype (i.e., low-level resistance to erythromycin, but sensitivity to clindamycin) and the presence of *mef* genes (*n* = 6/9) or the *ermTR* gene (*n* = 3/9) was statistically significant (*p* < 0.001 for both, Table 2). A further eight isolates, which were phenotypically macrolide sensitive, carried the *ermB* gene. The six *ermTR*+ isolates belonged to serotype V (*n* = 5) and serotype Ia (*n* = 1), and the D-test was positive in three cases. Among the 17 tetracycline sensitive isolates, 12 were fully macrolide sensitive and five had the M phenotype.

**Table 1:** Antibiotic susceptibility of the 96 GBS isolates

Antibiotic	MIC range [mg/L]	S%	I%	R%
Penicillin	0.016–0.064	100	–	0
Erythromycin	0.032- > 256	57.3	1.0	41.7
Clindamycin	0.064- > 256	64.6	–	35.4
Levofloxacin	0.5–16	96.9	–	3.1
Moxifloxacin	0.064–4	96.9	–	3.1
Tetracycline	0.125–128	17.7	0	82.3

**Table 2:** Association of M phenotype with the resistance genes

M phenotype	ermB			ermTR			mef A/E		
	+	–	*p* > 0.05	+	–	*p* < 0.05	+	–	*p* < 0.001
yes	1	8		3	6		6	3	
no	39	48		3	84		0	87	

### Serotypes and surface protein genes

For the 96 isolates in this study, the serotype prevalence in ranking order was the following: V (29.2%, *n* = 28), III (27.1%, *n* = 26), Ia (22.9%, *n* = 22), IV (10.4%, *n* = 10), II (5.2%, *n* = 5) and Ib (4.2%, *n* = 4), and finally one isolate proved to be non-typable. Always only one surface protein could be detected for each isolate; *rib* (32.3%) and *eps* (29.2%) were most common, followed by *alp2/3* (24.0%) and *alpC* (14.6%). A strong association was demonstrated between the surface protein genes and serotypes, such as *alp2/3* with type V, *rib* with type III, or *eps* with types Ia and IV (Table 3).

**Table 3:** Association between serotypes and surface protein genes

Serotype	Ia (*n* = 22)		Ib (*n* = 4)		II (*n* = 5)	III (*n* = 26)	IV (*n* = 10)			V (*n* = 28)			nt (*n* = 1)
Surface protein gene	eps	alpC	alpC	eps	alpC	rib	eps	alpC	rib	alp2/3	rib	alpC	rib
n	19	3	3	1	5	26	8	1	1	22	5	1	1

Serotypes Ia, Ib, and II were usually sensitive to macrolides, except for a type Ib strain, which was *linB* + *ermB* positive. Among serotype III isolates, both macrolide-resistant and sensitive strains were found, while serotype V was typically associated with macrolide resistance. Interestingly, although 6/10 serotype IV isolates were phenotypically macrolide sensitive, the *ermB* gene was detected in four of these. The highest levofloxacin and moxifloxacin MICs were measured with serotype II.

### Differences between isolates from specimens of the female or male urogenital tract

Of note, there were differences observed between the isolates deriving from the urogenital tract of female (*n* = 42) or male patients (*n* = 40). Whereas the prevalence of serotype V and IV was much higher in female specimens than in males (38.1% versus 20.0, and 11.9% versus 7.5%, respectively), the opposite was true for serotype III (21.4% versus 32.5%). As the surface protein genes showed a strong correlation with the serotypes, their presence also showed differences: meanwhile *Alp2/3* showed a prevalence of 28.6% versus 17.5% in the two gender groups; *AlpC* was present in 9.5 and 20.0%, respectively. However, none of these differences were statistically significant.

### Prevalence of *hvgA* and *bibA*

The ST-17 specific *hvgA* gene was detected in 21 isolates, which equals to 21.9% prevalence. Nineteen out of these were serotype III, one isolate was serotype IV and one was non-typeable. MLST analysis of the serotype III isolates confirmed that these were ST-17, while the serotype IV isolate proved to be ST-291, which differs from ST-17 in a single nucleotide in the *pheS* allele, therefore belongs to the CC-17 clonal complex as well. All remaining isolates (*n* = 75), belonging to several different serotypes, possessed the *bibA* gene (serotype V: *n* = 28, Ia: *n* = 22, IV: *n* = 9, III: *n* = 7, II: *n* = 5, Ib: *n* = 4).

### PFGE and MLST results

Although the GBS isolates showed a great level of diversity based on the PFGE patterns, major clones could be identified among the serotypes. Especially isolates of serotype V (possessing *alp2/3*) and Ia were shown to be clonal. The MLST results confirmed this: four representative isolates of the major serotype V PFGE cluster belonged to the ST-1 clone; and similarly, the tested serotype Ia isolates belonged to the ST-23 clone.

**Fig. 1:**
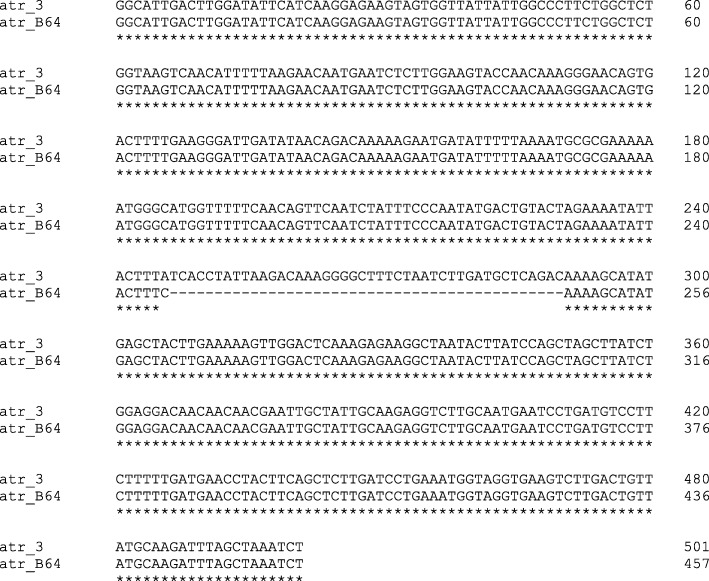
Sequence comparison of the *atr* locus of our isolate B64 (GenBank MG675237) and the *atr*-3 allele from the MLST database [40], showing the 44-bp deletion in the middle

However, there were two serotype V isolates (B48 and B64), which shared a common PFGE pattern and were *rib*+, but were highly dissimilar from members of the major serotype V clone (*alp2/3*+). B48 proved to belong to ST-110, but the other strain (B64) surprisingly contained a 44-bp deletion in the middle of the *atr* allele. Otherwise, its *atr* sequence showed identity with *atr*-3, which is present in ST-110 (Fig. 1). The *atr* gene fragment sequence of the isolate B64 has been deposited in the GenBank nucleotide sequence database under accession number MG675237.

Interestingly, the ST17 / serotype III isolates were quite heterogeneous based on the banding patterns. Sometimes different serotypes shared the same restriction pattern. We found one serotype IV isolate, which belonged to the ST-291 clone, which differs only in a single nucleotide in the *pheS* allele compared to ST-17, hence is a member of the CC-17 clonal complex. Similarly, one of the serotype IV isolates (*eps* +, macrolide-resistant) shared the same banding pattern of the major serotype V cluster, i.e. those belonging to ST-1, and the MLST analysis revealed that it was ST-196, which is a double-nucleotide variant of ST-1 (one nucleotide difference each in the *atr* and *glcK* alleles), and also part of CC-1. Two serotype IV isolates belonged to the ST-23 clone and one to the ST-24 clone, both being members of CC-23. Finally, two serotype Ib and one serotype II strain belonged to CC-10. A summary of the isolates of known sequence type is shown in Table 4.

**Table 4:** Summary of the isolates with known sequence type (*n* = 20)

Isolate	MLST	CC	hvgA	bibA	serotype	surface protein
B55	ST-17	CC-17	+	–	III	Rib
B89	ST-17	CC-17	+	–	III	Rib
B93	ST-291	CC-17	+	–	IV	Rib
B11	ST-19	CC-19	–	+	III	Rib
B92	ST-19	CC-19	–	+	III	Rib
B96	ST-12	CC10	–	+	II	AlpC
B73	ST-12	CC10	–	+	Ib	AlpC
B65	ST-8	CC10	–	+	Ib	AlpC
B72	ST-1	CC1	–	+	V	Alp2/3
B75	ST-1	CC1	–	+	V	Alp2/3
B21	ST-1	CC1	–	+	V	Alp2/3
B71	ST-1	CC1	–	+	V	Rib
B48	ST-110		–	+	V	Rib
B64	ST-110 - del		–	+	V	Rib
B81	ST-196	CC1	–	+	IV	Eps
B88	ST-24	CC-23	–	+	IV	AlpC
B101	ST-23	CC-23	–	+	IV	Eps
B16	ST-23	CC-23	–	+	Ia	Eps
B47	ST-23	CC-23	–	+	Ia	Eps
B90	ST-23	CC-23	–	+	Ia	Eps

## Discussion

**Fig. 2:**
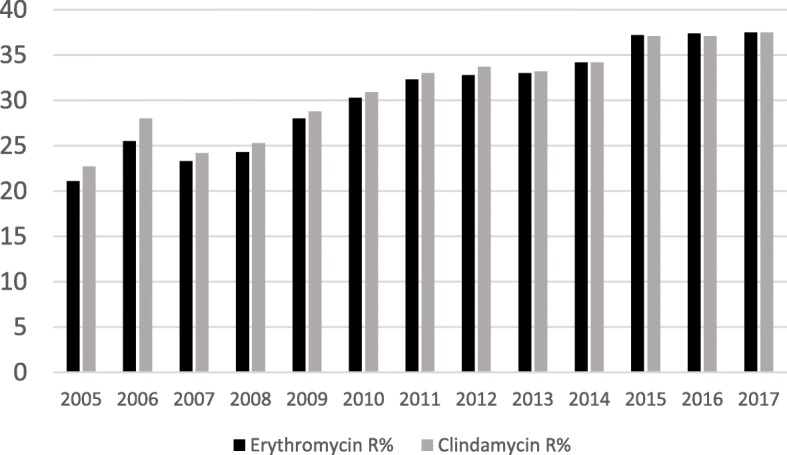
Erythromycin and clindamycin resistance data of Hungarian *S. agalactiae* isolates between 2005 and 2017 (10–18 thousand isolates yearly), as provided online by the National Public Health Center [41]

Whereas all isolates in this study were sensitive to penicillin, high resistance was observed to tetracycline and macrolides. Similar results are reported on the online surface of the Hungarian National Public Health Center (NPHC), where annual antibiotic sensitivity data are available (however, the origin of specimens – i.e. pregnancy screening or other - is unknown) [41]. As seen in Fig. 2, an increase in the macrolide resistance could be observed between 2005 and 2015, and the resistance rates stabilised in the last 3 years (last available data are from 2017).

The earliest sensitivity data of Hungarian GBS isolates were published in 1998, in a Hungarian journal [42], where the macrolide resistance of 362 isolates of different origin was only 5% (for both erythromycin and clindamycin). Newer data (from 2008 to 2010) were presented at the 17^th^ Alpe-Danube-Adria Congress on STD and Genital Dermatology (Budapest, 2011), and the analysis of 3382 GBS positive pregnant women revealed 29.1% erythromycin resistance in average (conference abstract, Ábrók et al., 2011). Interestingly, the authors observed higher resistance (38.0%) in the case of low-level GBS colonisation (*n* = 1058), and lower resistance (25.0%), in the case of high-level colonisation (*n* = 2324).

In the US, Gygax et al. found very similar values to these: out of 222 clinical GBS isolates, 38% were erythromycin-resistant and 21% clindamycin-resistant [32]. In Serbia, the overall erythromycin and clindamycin resistance rates were 23.1 and 21.3%, among 432 isolates deriving from both new-borns and pregnant / non-pregnant adults [43]. Regarding our six *ermTR* positive isolates, five of them were serotype V and one strain serotype 1a, and three strains had inducible resistance to clindamycin. This is in good concordance with the findings of Compain et al., where all six *ermTR* positive strains had iMLSB resistance and the two samples deriving from adults, were also serotype V and Ia [44].

This relatively high level of macrolide resistance detected by us is of worry, as in the case of penicillin allergy, clindamycin is the drug of choice against the colonising *S. agalactiae* for intrapartum antibiotic prophylaxis, to prevent early onset GBS infection of the neonate, according to the CDC guidelines [45]. If the isolate is (i) resistant to clindamycin, or (ii) sensitive to clindamycin but resistant to erythromycin, and the D-test is positive, then automatically vancomycin is suggested.

Furthermore, regarding genital GBS infection of non-pregnant women, C. Sonnex found that the patients treated with erythromycin failed to improve symptomatically, probably due to parallel presence of other microbes [12].

On the other hand, some countries report lower resistance rates. For example in a study conducted in Romania in 2009–2010, 91 GBS isolates from non-pregnant women had 14.3% erythromycin resistance [46]. Similar to this, erythromycin resistance was found to be 16.5% in Italy, from patients with non-invasive infections or carriers [23]. In Iceland, examining 145 GBS from invasive infections (between 1975 and 2014) revealed only 8.3 and 9.7% erythromycin and clindamycin resistance, respectively [47]. In this latter case, however, the 30–40 years old isolates might have decreased the overall resistance rates.

Very limited GBS serotyping data are available from Hungary in the literature. Only the above-mentioned study from 1998 [42] published serotypes of 362 strains (isolated in Szeged) and they observed the dominance of serotype III in the case of isolates from the genito-urinary tract, meanwhile in case of respiratory or skin specimens, serotypes I and II were more frequent. However, only types I-II-III were determined and out of the 362 strains, 22 were negative for these types (these could probably be serotype V or IV). Since that study, until now, no serotyping data have been published from Hungary.

In the current project, serotypes V (29.2%), III (27.1%) and Ia (22.9%) were predominant. This corresponds well with data from other European countries, except for the prevalence of serotype II. In our study, only five isolates were serotype II (=5.2%), while this type is better represented in some studies. In Romania, which is bordered to Hungary, serotypes III (*n* = 27), II (*n* = 21), Ia (*n* = 20) and V (*n* = 18) were most frequent among 91 GBS from non-pregnant women [46]. Almost the same results were obtained in a German study from 2006, where the serotype prevalence among 75 GBS isolated from both pregnant and non-pregnant women was the following: serotype III (*n* = 21), II (*n* = 16), Ia (*n* = 13) and V (*n* = 12) [48]. A recent Polish study investigated 353 GBS from pregnancy screening, and they also found serotype III to be predominant (35%), followed by Ia (20%), V (17%) and II (15%) [24]. Gherardi et al. investigated GBS isolates from both clinical and colonising origin in Italy, and they found that meanwhile serotype III dominated in the colonising population, serotype V was most frequently found among GBS causing non-invasive infections [23]. In Ireland, serotypes V and II were best represented among 31 GBS deriving from invasive infections of non-pregnant adults, with eight strains each [49].

Based on the data found in the literature, we can conclude that meanwhile usually serotype III dominates among *S. agalactiae* deriving from pregnancy screening, serotype V could be more frequently seen in infections of non-pregnant adults. Serotype V has been detected increasingly since the 1990s and has become especially important in invasive infections of non-pregnant adults [50].

A strong correlation between serotypes and surface proteins was established in our study. This is also well known from the literature [20, 23, 24, 46, 48].

There are ongoing vaccine developments in different clinical trials. The vaccine candidates are either capsular polysaccharides (conjugated or not) or exposed surface proteins such as AlphaC and Rib [51]. Therefore the results provided by the current study could contribute to a more specialised regional GBS vaccine construction.

More than one fifth (21.9%) of our isolates belong to the ST-17 hypervirulent clone. This is a remarkable proportion of *S. agalactiae* from entirely non-pregnant patients, as this sequence type is typically associated with neonatal infections, accounting for >80% of LOD cases [52]. For instance, a much lower rate was found in Romania (6.1%, 9 out of 148 GBS), including isolates from both pregnant and non-pregnant women [46]. According to Martins et al., ST-17 was responsible only for a minority of invasive infections of non-pregnant adults in Portugal [53]. Serbian authors found that whereas ST-17 was isolated in 93.3% of LOD cases, its prevalence was only 5.7% in non-pregnant adults [43]. As Tien et al. also concluded in their study, CC17 appears to be adapted to neonates, while CC12 and CC1 rather to adults. These could be explained either by differences in host immune response or allelic exchange [50].

We found one serotype IV isolate belonging to the ST-196 clone (=CC1) and another one to the ST-291 (=CC17), which is a single locus variant of ST-17. One serotype IV isolate belonged to ST-23, which is a common clone among GBS infections, however, usually comprising of type Ia or type III strains [24, 43, 47, 50, 54]. A Spanish study found two similar strains among 107 GBS collected from menopausal women [55]. The emergence of new serotype IV clones were reported in recent years from several countries [56]. This increase might be explained by genetic recombination events, such as capsular switching or acquisition of the HvgA adhesin [52]. A particular increase of serotype IV was observed in the frame of a large-scale study in Portugal, where the increase rate was from 1.4% in 2006 to 19.6% in 2012 [57]. In their study, nine of the 89 (=10.1%) serotype IV isolates proved to belong to ST-291. The same ST-291/ serotype IV was also reported from Taiwan [50]. Furthermore, serotype IV isolates displaying either ST-291 (CC17, *n* = 3) or ST-196 (CC1, *n* = 2) were identified in France, among 965 invasive GBS, collected between 2007 and 2011 [58] and in the US as well [59]. One ST-196 isolate was also found in Italy, already in the period of 2002–2005 [23].

## Conclusions

This is the first report of *Streptococcus agalactiae* deriving from non-pregnant adults from Hungary. The macrolide resistance of the isolates was higher than observed in other countries and it is increasing slowly over the years. The presence of silent *ermB* genes (i.e. detected by PCR in isolates phenotypically macrolide sensitive) is of worry, as (i) these strains could serve as reservoirs for horizontal resistance gene transfer and (ii) expression of these genes triggered by exposure to macrolides could lead to unforeseen therapeutic failure.

Furthermore, this is the first study in the international literature providing serotyping and genotyping data from Hungarian GBS isolates. In our collection of 96 strains, serotype V and III were most prevalent with 29 and 27%, respectively. This is in good correlation with the fact that serotype V is becoming more frequent among infections of non-pregnant adults. We found an unusual serotype V strain, which had a 44-bp deletion in the *atr* allele compared to that of ST-110. Notably, an unusually high proportion (21.9%) of our isolates belongs to the ST-17 hypervirulent clone, which is normally responsible for invasive infections of neonates. The patients of the current study, GBS positive men and women, could be sources of further spread of this dangerous clone. Finally, although we found only 10.4% serotype IV isolates, some of these belong to emerging clonal lineages [52, 56].

The study has the following limitations: (i) the GBS isolates included in the study were isolated almost one decade ago, (ii) only one third of the GBS isolates (96 out of 296) obtained in the study period at the laboratory was included in the study, (iii) only a subset of 20 isolates was analysed by MLST.

## Data Availability

All data generated or analysed during this study are included in this published article.

## Abbreviations

LOD: Late onset disease
EOD: Early onset disease
HvgA: Hypervirulent GBS adhesion
BibA: GBS immunogenic bacterial adhesin
NPHC: Hungarian National Public Health Center
STD: Sexually transmitted diseases
GBS: Group B streptococcus
PFGE: Pulsed-field gel electrophoresis
PCR: Polymerase chain reaction
ST: Sequence type
CC: Clonal complex
MLST: Multi-locus sequence typing
EUCAST: European Committee for Antimicrobial Susceptibility Testing
*S. pneumoniae*: *Streptococcus pneumoniae*
MIC: Minimal inhibitory concentration
S, I, R: Sensitive, intermediate, resistant
CDC: Centers for Disease Control and Prevention

## References

[1] Schuchat A (1999). Group B streptococcus. Lancet.

[2] Baker CJ, Stevens DL, Kaplan EL (2000). Group B streptococcal infections. Streptococcal infections.

[3] Melin P (2011). Neonatal group B streptococcal disease: from pathogenesis to preventive strategies. Clin Microbiol Infect.

[4] Schuchat A (1998). Epidemiology of group B streptococcal disease in the United States: shifting paradigms. Clin Microbiol Rev.

[5] Shet A, Ferrieri P (2004). Neonatal & maternal group B streptococcal infections: a comprehensive review. Indian J Med Res.

[6] Skoff TH, Farley MM, Petit S, Craig AS, Schaffner W, Gershman K (2009). Increasing burden of invasive group B streptococcal disease in nonpregnant adults, 1990-2007. Clin Infect Dis.

[7] Le Doare K, Heath PT (2013). An overview of global GBS epidemiology. Vaccine.

[8] Phares CR, Lynfield R, Farley MM, Mohle-Boetani J, Harrison LH, Petit S (2008). Epidemiology of invasive group B streptococcal disease in the United States, 1999-2005. JAMA.

[9] Blancas D, Santin M, Olmo M, Alcaide F, Carratala J, Gudiol F (2004). Group B streptococcal disease in nonpregnant adults: incidence, clinical characteristics, and outcome. Eur J Clin Microbiol Infect Dis.

[10] Edwards MS, Baker CJ (2005). Group B streptococcal infections in elderly adults. Clin Infect Dis.

[11] Honig E, Mouton JW, van der Meijden WI (2002). The epidemiology of vaginal colonisation with group B streptococci in a sexually transmitted disease clinic. Eur J Obstet Gynecol Reprod Biol.

[12] Sonnex C (2013). Genital streptococcal infection in non-pregnant women: a case-note review. Int J STD AIDS.

[13] Adam A, Pal Z, Terhes G, Szucs M, Gabay ID, Urban E (2018). Culture- and PCR-based detection of BV associated microbiological profile of the removed IUDs and correlation with the time period of IUD in place and the presence of the symptoms of genital tract infection. Ann Clin Microbiol Antimicrob.

[14] Ross PW, Cumming CG (1982). Group B streptococci in women attending a sexually transmitted diseases clinic. J Inf Secur.

[15] Manning SD, Neighbors K, Tallman PA, Gillespie B, Marrs CF, Borchardt SM (2004). Prevalence of group B streptococcus colonization and potential for transmission by casual contact in healthy young men and women. Clin Infect Dis.

[16] El Beitune P, Duarte G, Maffei CM, Quintana SM, Rosa AC, Silva E, Nogueira AA. Group B Streptococcus carriers among HIV-1 infected pregnant women: prevalence and risk factors. Eur J Obstet Gynecol Reprod Biol 2006;128(1–2):54–58. 10.1016/j.ejogrb.2006.02.017 16621230

[17] Baker CJ, Barrett FF (1974). Group B streptococcal infections in infants. The importance of the various serotypes. JAMA.

[18] Spellerberg B (2000). Pathogenesis of neonatal *Streptococcus agalactiae* infections. Microbes Infect.

[19] Lindahl G, Stålhammar-Carlemalm M, Areschoug T (2005). Surface proteins of *Streptococcus agalactiae* and related proteins in other bacterial pathogens. Clin Microbiol Rev.

[20] Creti R, Fabretti F, Orefici G, von Hunolstein C (2004). Multiplex PCR assay for direct identification of group B streptococcal alpha-protein-like protein genes. J Clin Microbiol.

[21] Lachenauer CS, Creti R, Michel JL, Madoff LC (2000). Mosaicism in the alpha-like protein genes of group B streptococci. Proc Natl Acad Sci U S A.

[22] Kong F, Gowan S, Martin D, James G, Gilbert GL (2002). Molecular profiles of group B streptococcal surface protein antigen genes: relationship to molecular serotypes. J Clin Microbiol.

[23] Gherardi G, Imperi M, Baldassarri L, Pataracchia M, Alfarone G, Recchia S (2007). Molecular epidemiology and distribution of serotypes, surface proteins, and antibiotic resistance among group B streptococci in Italy. J Clin Microbiol.

[24] Brzychczy-Włoch M, Gosiewski T, Bodaszewska-Lubas M, Adamski P, Heczko PB (2012). Molecular characterization of capsular polysaccharides and surface protein genes in relation to genetic similarity of group B streptococci isolated from polish pregnant women. Epidemiol Infect.

[25] Weisner AM, Johnson AP, Lamagni TL, Arnold E, Warner M, Heath PT (2004). Characterization of group B streptococci recovered from infants with invasive disease in England and Wales. Clin Infect Dis.

[26] Musser JM, Mattingly SJ, Quentin R, Goudeau A, Selander RK (1989). Identification of a high-virulence clone of type III *Streptococcus agalactiae* (group B Streptococcus) causing invasive neonatal disease. Proc Natl Acad Sci U S A.

[27] Bisharat N, Crook DW, Leigh J, Harding RM, Ward PN, Coffey TJ (2004). Hyperinvasive neonatal group B streptococcus has arisen from a bovine ancestor. J Clin Microbiol.

[28] Lamy MC, Dramsi S, Billoët A, Réglier-Poupet H, Tazi A, Raymond J (2006). Rapid detection of the “highly virulent” group B Streptococcus ST-17 clone. Microbes Infect.

[29] Tazi A, Disson O, Bellais S, Bouaboud A, Dmytruk N, Dramsi S (2010). The surface protein HvgA mediates group B streptococcus hypervirulence and meningeal tropism in neonates. J Exp Med.

[30] Santi I, Scarselli M, Mariani M, Pezzicoli A, Masignani V, Taddei A (2007). BibA: a novel immunogenic bacterial adhesin contributing to group B Streptococcus survival in human blood. Mol Microbiol.

[31] EUCAST. European Committee on Antimicrobial Susceptibility Testing. Breakpoint tables for interpretation of MICs and zone diameters, version 9.0, 2019. http://www.eucast.org/clinical_breakpoints/ Accessed 16 July 2019.

[32] Gygax SE, Schuyler JA, Kimmel LE, Trama JP, Mordechai E, Adelson ME (2006). Erythromycin and clindamycin resistance in group B streptococcal clinical isolates. Antimicrob Agents Chemother.

[33] Bozdogan B, Berrezouga L, Kuo MS, Yurek DA, Farley KA, Stockman BJ (1999). A new resistance gene, linB, conferring resistance to lincosamides by nucleotidylation in *Enterococcus faecium* HM1025. Antimicrob Agents Chemother.

[34] Seppälä H, Skurnik M, Soini H, Roberts MC, Huovinen P (1998). A novel erythromycin resistance methylase gene (ermTR) in *Streptococcus pyogenes*. Antimicrob Agents Chemother.

[35] Oster P, Zanchi A, Cresti S, Lattanzi M, Montagnani F, Cellesi C (1999). Patterns of macrolide resistance determinants among community-acquired *Streptococcus pneumoniae* isolates over a 5-year period of decreased macrolide susceptibility rates. Antimicrob Agents Chemother.

[36] Poyart C, Tazi A, Réglier-Poupet H, Billoët A, Tavares N, Raymond J (2007). Multiplex PCR assay for rapid and accurate capsular typing of group B streptococci. J Clin Microbiol.

[37] Imperi M, Pataracchia M, Alfarone G, Baldassarri L, Orefici G, Creti R (2010). A multiplex PCR assay for the direct identification of the capsular type (Ia to IX) of *Streptococcus agalactiae*. J Microbiol Methods.

[38] Benson JA, Ferrieri P (2001). Rapid pulsed-field gel electrophoresis method for group B streptococcus isolates. J Clin Microbiol.

[39] van Belkum A, Tassios PT, Dijkshoorn L, Haeggman S, Cookson B, Fry NK (2007). Guidelines for the validation and application of typing methods for use in bacterial epidemiology. Clin Microbiol Infect.

[40] MLST Multi Locus Sequence Typing website. https://pubmlst.org/sagalactiae/. Accessed 15 July 2019.

[41] National Bacteriological Surveillance Management Team. NBS Annual reports. National Public Health Center, Budapest, Hungary. http://oek.hu/oek.web. Accessed 7 July 2019.

[42] Dósa E, Urbán E, Nagy E (1999). Serotype distribution of *Streptococcus agalactiae* strains originating from different clinical materials [in Hungarian]. Infektológia és klinikai mikrobiológia.

[43] Gajic I, Plainvert C, Kekic D, Dmytruk N, Mijac V, Tazi A (2018). Molecular epidemiology of invasive and non-invasive group B Streptococcus circulating in Serbia. Int J Med Microbiol.

[44] Compain F, Hays C, Touak G, Dmytruk N, Trieu-Cuot P, Joubrel C (2014). Molecular characterization of *Streptococcus agalactiae* isolates harboring small erm (T)-carrying plasmids. Antimicrob Agents Chemother.

[45] Verani JR, McGee L, Schrag SJ, Centers for Disease Control and Prevention (CDC) (2010). Prevention of perinatal group B streptococcal disease--revised guidelines from CDC, 2010. MMWR Recomm Rep.

[46] Usein CR, Grigore L, Georgescu R, Cristea V, Baltoiu M, Straut M (2012). Molecular characterization of adult-colonizing *Streptococcus agalactiae* from an area-based surveillance study in Romania. Eur J Clin Microbiol Infect Dis.

[47] Björnsdóttir ES, Martins ER, Erlendsdóttir H, Haraldsson G, Melo-Cristino J, Kristinsson KG (2016). Changing epidemiology of group B streptococcal infections among adults in Iceland: 1975–2014. Clin Microbiol Infect.

[48] Brimil N, Barthell E, Heindrichs U, Kuhn M, Lütticken R, Spellerberg B (2006). Epidemiology of *Streptococcus agalactiae* colonization in Germany. Int J Med Microbiol.

[49] Meehan M, Cunney R, Cafferkey M (2014). Molecular epidemiology of group B streptococci in Ireland reveals a diverse population with evidence of capsular switching. Eur J Clin Microbiol Infect Dis.

[50] Tien N, Ho CM, Lin HJ, Shih MC, Ho MW, Lin HC (2011). Multilocus sequence typing of invasive group B Streptococcus in central area of Taiwan. J Microbiol Immunol Infect.

[51] Heath PT (2016). Status of vaccine research and development of vaccines for GBS. Vaccine..

[52] Shabayek S, Spellerberg B (2018). Group B streptococcal colonization, molecular characteristics, and epidemiology. Front Microbiol.

[53] Martins ER, Melo-Cristino J, Ramirez M (2012). The Portuguese Group for the Study of streptococcal infections. Dominance of serotype Ia among group B streptococci causing invasive infections in nonpregnant adults in Portugal. J Clin Microbiol.

[54] Martins ER, Pedroso-Roussado C, Melo-Cristino J, Ramirez M, The Portuguese Group for the Study of Streptococcal Infections (2017). Causing Neonatal Infections in Portugal (2005–2015): Diversification and Emergence of a CC17/PI-2b Multidrug Resistant Sublineage. Front Microbiol.

[55] Moltó-García B, MeC L-M, Cuadros-Moronta E, Rodríguez-Granger J, Sampedro-Martínez A, Rosa-Fraile M (2016). Molecular characterization and antimicrobial susceptibility of hemolytic *Streptococcus agalactiae* from post-menopausal women. Maturitas.

[56] Campisi E, Rinaudo CD, Donati C, Barucco M, Torricelli G, Edwards MS (2016). Serotype IV *Streptococcus agalactiae* ST-452 has arisen from large genomic recombination events between CC23 and the hypervirulent CC17 lineages. Sci Rep.

[57] Florindo C, Damiao V, Silvestre I, Farinha C, Rodrigues F, Nogueira F (2014). Epidemiological surveillance of colonising group B Streptococcus epidemiology in the Lisbon and Tagus Valley regions, Portugal (2005 to 2012): emergence of a new epidemic type IV/clonal complex 17 clone. Euro Surveill.

[58] Bellais S, Six A, Fouet A, Longo M, Dmytruk N, Glaser P (2012). Capsular switching in group B Streptococcus CC17 hypervirulent clone: a future challenge for polysaccharide vaccine development. J Infect Dis.

[59] Ferrieri P, Lynfield R, Creti R, Flores AE (2013). Serotype IV and invasive group B Streptococcus disease in neonates, Minnesota, USA, 2000-2010. Emerg Infect Dis.

